# End-Stage Renal Disease-Associated Gut Bacterial Translocation: Evolution and Impact on Chronic Inflammation and Acute Rejection After Renal Transplantation

**DOI:** 10.3389/fimmu.2019.01630

**Published:** 2019-08-16

**Authors:** Clémence Carron, Jean-Paul Pais de Barros, Emilie Gaiffe, Valérie Deckert, Hanane Adda-Rezig, Caroline Roubiou, Caroline Laheurte, David Masson, Dominique Simula-Faivre, Pascale Louvat, Bruno Moulin, Luc Frimat, Philippe Rieu, Christiane Mousson, Antoine Durrbach, Anne-Elisabeth Heng, Philippe Saas, Didier Ducloux, Laurent Lagrost, Jamal Bamoulid

**Affiliations:** ^1^Univ. Bourgogne Franche-Comté, INSERM, EFS BFC, UMR1098, Interactions Hôte-Greffon-Tumeur/Ingénierie Cellulaire et Génique, Fédération Hospitalo-Universitaire INCREASE, LabEx LipSTIC, Besançon, France; ^2^INSERM, LabEx LipSTIC, Univ. Bourgogne Franche-Comté, LNC UMR1231, Dijon, France; ^3^FHU INCREASE, Besançon, France; ^4^INSERM CIC-1431, LabEx LipSTIC, Clinical Investigation Center in Biotherapy, University Hospital of Besançon, Fédération Hospitalo-Universitaire INCREASE, Besançon, France; ^5^Department of Nephrology, Dialysis, and Renal Transplantation, University Hospital of Besançon, Besançon, France; ^6^Plateforme de BioMonitoring, EFS Bourgogne Franche-Comté, Besançon, France; ^7^CHU Dijon, Biochimie et Service de la Recherche, Dijon, France; ^8^Department of Nephrology, CHU Strasbourg, Dialysis, and Renal Transplantation, Strasbourg, France; ^9^Department of Nephrology, CHU Nancy, Dialysis, and Renal Transplantation, Nancy, France; ^10^Department of Nephrology, CHU Reims, Dialysis, and Renal Transplantation, Reims, France; ^11^Department of Nephrology, CHU Dijon, Dialysis, and Renal Transplantation, Dijon, France; ^12^Department of Nephrology, CHU Kremlin-Bicêtre, Dialysis, and Renal Transplantation, Le Kremlin-Bicêtre, France; ^13^Department of Nephrology, CHU Clermont-Ferrand, Dialysis, and Renal Transplantation, Clermont-Ferrand, France

**Keywords:** gut bacterial translocation, lipopolysaccharides, chronic inflammation, cholesterol, acute rejection, kidney transplantation

## Abstract

Chronic inflammation in end-stage renal disease (ESRD) is partly attributed to gut bacterial translocation (GBT) due to loss of intestinal epithelium integrity. Increased levels of circulating lipopolysaccharide (LPS) –a surrogate marker of GBT– contribute to maintain a chronic inflammatory state. However, circulating LPS can be neutralized by lipoproteins and transported to the liver for elimination. While ESRD-associated GBT has been widely described, less is known about its changes and impact on clinical outcome after kidney transplantation (KT). One hundred and forty-six renal transplant recipients with serum samples obtained immediately before and 1 year after transplantation (1-Year post KT) were included. Intestinal epithelium integrity (iFABP), total LPS (by measuring 3-hydroxymyristate), LPS activity (biologically active LPS measured by the LAL assay), inflammatory biomarkers (sCD14 and cytokines), lipoproteins and LPS-binding proteins (LBP and phospholipid transfer protein [PLTP] activity) were simultaneously measured. At 1-Year post KT, iFABP decreased but remained higher than in normal volunteers. Total LPS concentration remained stable while LPS activity decreased. Inflammation biomarkers decreased 1-Year post KT. We concomitantly observed an increase in lipoproteins. Higher sCD14 levels before transplantation was associated with lower incidence of acute rejection. Although GBT remained stable after KT, the contemporary increase in lipoproteins could bind circulating LPS and contribute concomitantly to neutralization of LPS activity, as well as improvement in ESRD-associated chronic inflammation. Chronic exposure to LPS in ESRD could promote endotoxin tolerance and explain why patients with higher pre-transplant sCD14 are less prompt to develop acute rejection after transplantation.

## Introduction

End-stage renal disease (ESRD) is associated with elevated plasma concentrations of pro-inflammatory cytokines and activated/exhausted leukocytes (1–3). This state of persistent low-grade inflammation plays a major role in the progression of chronic kidney disease (CKD), and has been recognized as a promoter for cardiovascular disease (CVD) in CKD (4, 5). This chronic inflammation results from the persistence of a causative stimulus stemming from several dialysis-related factors, like membrane bio-incompatibility, and non–dialysis-related factors like infections, oxidative stress, accumulation of uremic toxins, comorbidities, genetic factors, diet (4), or more recently described, gut bacterial translocation (GBT) (6). GBT has been attributed to a loss of gut epithelial barrier integrity (due to accumulation of uremic toxins, metabolites and urea) and intestinal hypoperfusion (7), and GBT is potentially aggravated by intra-dialytic and post-dialytic hypotension (8, 9). Damage to the integrity of the gut epithelial barrier allows bacteria and their products, especially lipopolysaccharides (LPS)—a potent immune stimulating factor-, to translocate from the intestinal lumen into the peripheral blood (10). This phenomenon is also known as endotoxemia. LPS-binding protein (LBP) carries and contributes to the transfer of LPS on innate immune cells expressing CD14 and Toll-Like Receptor-4 (TLR-4). The binding of the LPS to TLR-4 stimulates the MyD88 and TRIF signaling pathways, involved in pro-inflammatory cytokine release and subsequent chronic inflammation (11). CD14 also exists as a soluble form (sCD14) able to interact directly with LPS and to participate to the cell activation. Moreover, sCD14, like LBP, also facilitates the transfer of LPS to HDL, resulting in the neutralization of LPS. Indeed, biological activity of circulating LPS may be neutralized by lipoproteins and, thanks to the “reverse LPS transport” pathway, driven to the liver. Earlier studies reported that phospholipid transfer protein (PLTP) may play a key role in mediating lipoprotein binding and neutralization of LPS (12).

The LAL assay—using lysate of horseshoe crab amebocytes, which enzymatically interacts with endotoxins—is currently the most frequent method for detection of circulating LPS (13, 14) and assessment of GBT. However, the LAL assay better reflects LPS activity than a real quantification of the total LPS mass concentration (13). Moreover, Bohrer et al. (15) showed that peritoneal dialysis fluids and hemodialysis concentrates can interfere with the LAL assay. Until now, effective blood measurements of LPS are missing in clinical studies (13). Quantitative LPS analysis is now available, using spectrometry coupled to high performance liquid chromatography (16). This method measures total circulating mass of LPS, including bound and unbound LPS, and not only LPS activity (i.e., unbound LPS able to activate the LAL assay).

Hence, we aimed to shed new light on ESRD- associated GBT, combining measurement of total LPS quantity (16) and LAL assay. In association, we explored gut epithelial barrier integrity using a marker of enterocyte damages (17), i.e., intestinal fatty acid binding protein (iFABP); induced inflammation, by measuring circulating sCD14 levels, as well as inflammatory cytokines, and LPS elimination pathway. While ESRD-associated GBT has been widely described, less is known about its changes after kidney transplantation (KT). Therefore, we explored in a second step the evolution of GBT and inflammation after weaning of dialysis and improvement in renal function following KT.

Our main hypothesis is that renal function recovery enables a decrease in uremic toxin concentrations, as well as an improvement in renal and intestinal perfusion, and enhances lipoprotein metabolism with an increase in circulating cholesterol-high density lipoproteins (C-HDL). All these parameters may influence GBT and related inflammation.

## Materials and Methods

### Patient Population and Samples

Research has been conducted on biological samples collected in 146 incident renal transplant recipients (RTR) from the ORLY-Est study (18). The ORLY-Est study is a prospective observational study designed to assess immunologic factors predictive of post-transplant atherosclerotic events (inclusion and exclusion criteria are given in Supplementary Table 1). Seven French transplant centers (Besançon, Dijon, Nancy, Kremlin-Bicetre, Clermont-Ferrand, Reims, and Strasbourg) participate in this study. To date, 965 patients have been enrolled. Samples were collected and sent to the Biomonitoring Platform (CIC-BT506, EFS Besançon, France) for processing and storage. As we hypothesized a relationship between GBT and acute rejection (AR), we included more patients with AR: each patient with a history of AR was matched for the main demographical characteristics, with 1 or 2 patients free from AR (Supplementary Table 2). The 57 first incident renal transplant patients from the ORLY-Est cohort with a history of AR and with available samples were matched to 1 or 2 patients without history of AR from the same period of inclusion according to the demographic characteristics detailed in Supplementary Table 2. Hence, 146 patients were included (57 AR and 89 without AR). For each patient, blood samples were collected prior to transplantation (ESRD) and 1 year after. Sera were isolated by centrifugation and cryopreserved. Samples collection was performed after regulatory approval by the French ministry of health (agreement numbers # DC-2008-713, June 2009 and DC-2015-2373, October 2015). The ethic committee of Franche-Comté has approved the study (2008). Patients enrolled in the ORLY-EST study gave their written informed consent. Clinical data were prospectively collected. For details, see the Methods in the Supporting Information section. Sera were also collected from 11 healthy anonymous volunteers at the Etablissement Français du Sang (EFS, Besançon, France). Most patients were given CNI, corticosteroids and mycophenolic acid at time of GBT measurements and the maintenance immunosuppressive regimen was comparable between patients enrolled in the present study and the rest of the ORLY-Est cohort (Table 1). At 1-year, most patients had no or tapered corticosteroid doses (5–10 mg according to the center). Levels of CNI and mycophenolic acid were managed by each center according to their own immunosuppression protocols.

**Table 1:** Description of general characteristics of the study RTR population and total RTR population.

**Variable**	**Study population**	**Total RTR cohort**	***p***
*n*	146	788	
Age, mean in years (range)	50.0 (20–74)	52.4 (18–84)	**0.040**
Sex ratio (H/F)	1.60	1.75	0.656
BMI (kg/m^2^), mean	25.3	25.6	0.483
Dialysis *n*. (%)	133 (91.1)	711 (90.2)	0.621
HD *n*. (%)	94 (70.7)	540 (75.9)	0.419
DP *n*. (%)	28 (21.1)	144 (20.3)	0.747
DP/HD *n*. (%)	10 (7.5)	27 (3.8)	**0.047**
Duration of dialysis, mean in months (range)	34.2 (0-219)	40.2 (0-432)	0.107
First renal transplantation *n*. (%)	135 (92.5)	718 (91.1)	0.595
Presence of anti-HLA antibodies *n*. (%)	46 (31.5)	241 (30.6)	0.891
CMV exposure *n*. (%)	117 (80.1)	662 (84.0)	0.752
Diabetes before transplantation *n*. (%)	22 (15.0)	154 (19.5)	0.186
Anti-CD25 *n*. (%)	89 (61.0)	446 (56.6)	0.495
ATG *n*. (%)	57 (39.0)	230 (29.2)	**0.042**
Corticosteroid *n*. (%)	134 (91.8)	721 (91.5)	0.910
Tacrolimus *n*. (%)	104 (71.2)	511 (64.8)	0.135
Ciclosporin *n*. (%)	33 (22.6)	231 (29.3)	0.098
Sirolimus/Everolimus *n*. (%)	4 (2.7)	20 (2.5)	0.888
Mycophenolate mofetil/Mycophenolic acid *n*. (%)	140 (95.9)	758 (96.2)	0.574
Azathioprine *n*. (%)	3 (2.1)	1 (0.1)	**0.001**

*n, number; BMI, body mass index; HD, hemodialysis; DP, peritoneal dialysis; CMV, Cytomegalovirus; ATG, Anti-thymocyte globulins. Bold values mean statistically significant values*.

### Immunostaining

Absolute numbers of CD4^+^ and CD8^+^ T cells were determined on fresh samples by a single platform flow cytometry approach using the TetraCXP method, Flow-Count fluorospheres, and FC500 cytometer (Beckman Coulter, Villepinte, France) according to the manufacturer's recommendations. Pro-inflammatory monocytes were stained on fresh samples with the following conjugated antibodies directed against CD45 (APC, Pharmingen), CD14 (ECD, Immunotech), CD16 (PC-7, Immunotech), HLA-DR (FITC, Pharmingen), CD86 (PE, Immunotech) according to the manufacturer's recommendations. Staining was analyzed on FC500 cytometer (Beckman Coulter, Villepinte, France). Pro-inflammatory monocyte percentages and counts were performed on 74 samples.

### Endotoxemia

Endotoxin levels were evaluated by both circulating LPS activity and total LPS quantity measured as described in Pais de Barros et al. (16). Biological activity of LPS was quantified in serum of 89 RTR by the end-point chromogenic *Limulus amebocyte* lysate (LAL) assay (QCL-1000 kit; Lonza, Walkersville, MD USA) which gives a magenta color when positive. Briefly, 50 μl of diluted plasma (1:20 dilution in endotoxin-free water) were dispensed in each well of a 96-well plate. At the initial time point, 50 μl of the LAL reagent were added to each well. The plate was shaken and incubated at 37°C for 10 min. Then, 100 μl of chromogenic substrate warmed to 37°C was added to each well and incubation was extended for an additional 6 min at 37°C. The reaction was stopped by adding 100 μl of a 25% solution of glacial acetic acid. Absorbance was measured at 405 nm on a spectrophotometer (Victor3, Perkin Elmer). Total LPS concentration was determined in serum of all 146 RTR by direct quantitation of 3-hydroxytetradecanoic acid (3-hydroxymyristate or 3HM) by high performance liquid chromatography coupled with mass spectrometry (HPLC/MS/MS) (16). 3-HM molecules are indeed bound to the lipid A motif of LPS and allow us to quantify circulating total LPS (16).

### Soluble Factors

iFABP, LBP, and sCD14 were measured in serum using enzyme-linked immunosorbent assay kits, according to the manufacture's recommendations. iFABP serum levels were diluted 1:3 and measured in 146 RTR with Hycult Biotech kit (Uden, Netherlands). LBP serum levels were diluted 1:1000 and measured in 57 RTR with Hycult Biotech kit (Uden, Netherlands). Soluble CD14 serum levels were diluted 1:400 and measured in 146 RTR with Quantikine ELISA kit (R&D Systems, Minneapolis, MN).

### Pro-Inflammatory Cytokines

The concentrations of IL-1β, IL-6, IL-8, and TNF-α were determined in serum of 89 RTR by using a Milliplex MAP Human Cytokine/Chemokine Magnetic Bead Panel kit (Millipore, Billerica, MA). The assays were performed according to the manufacturer's instructions. Standards and samples were analyzed on a LuminexR® apparatus (Bio-Plex 200, BioRad, München, Germany) using the BioPlex Manager Software (Version 5, BioRad, Hercules, CA).

### PLTP Activity and Lipoproteins

PLTP activity was measured in serum of 89 RTR using a commercially available fluorescence activity assay from Roar Biomedical (New York, NY, USA), according to the manufacturer's instructions. This fluorimetric assay measures the transfer (unquenching) of fluorescent phospholipids from donor to acceptor synthetic liposomes. Phospholipid transfer rates were calculated using the initial slope of the phospholipid transfer curve, and were expressed as initial phospholipid transfer rate (i.e., nmol/h/ml serum). Serum lipoproteins were assayed in 89 RTR using commercially available kits (Cholesterol, HDL-cholesterol and Triglycerides, Thermo Fisher Scientific, Finland) on an Indiko Clinical Chemistry analyzer (Thermo Fisher Scientific, Finland) according to the manufacturer's instructions.

### Clinical Outcomes

Clinical outcomes occurring during the year period following KT were prospectively collected and registered by an independent committee. Definitions are available in the supplementary text in the Supporting Information section. CMV disease, opportunistic infections, severe bacterial infections, acute rejection (AR), new onset diabetes mellitus (NODAT), atherosclerotic events, graft loss, death-censored allograft survival and death were defined prospectively in the ORLY-Est study.

### Statistical Analysis

Data were expressed as means ± SD, median with 25 and 75th percentiles or as percentages. Study values at transplant and 1 year after were compared using Wilcoxon paired *t*-test, others values using Mann-Whitney unpaired *t*-test. Spearman rank test was used to determine correlations. Discontinuous variables were compared using Khi 2 test. All *p*-values below 0.05 were considered statistically significant. All statistical analyses were performed using GraphPad Prism 6 (GraphPad Software, San Diego, CA) and SPSS Statistics 23.0 (IBM, Chicago, USA).

## Results

### Population Characteristics

One hundred and forty-six ESRD patients from the ORLY-Est study were included. We first compared this population to the ORLY-Est population of patients not included in this analysis (PNI, *n* = 788) (Table 1) and to normal volunteers (NV, *n* = 11). The studied population was younger than PNI (50.0 vs. 52.4 y-o; *p* = 0.032) but older than NV (50.0 vs. 38.7 y-o; *p* = 0.004). However, there was no correlation between biomarkers and patients' age (data not shown) and no data clearly supports any influence of age on intestinal permeability (19–21), especially in ESRD.

### Gut Bacterial Translocation and Chronic Inflammation in ESRD

**Figure 1:**
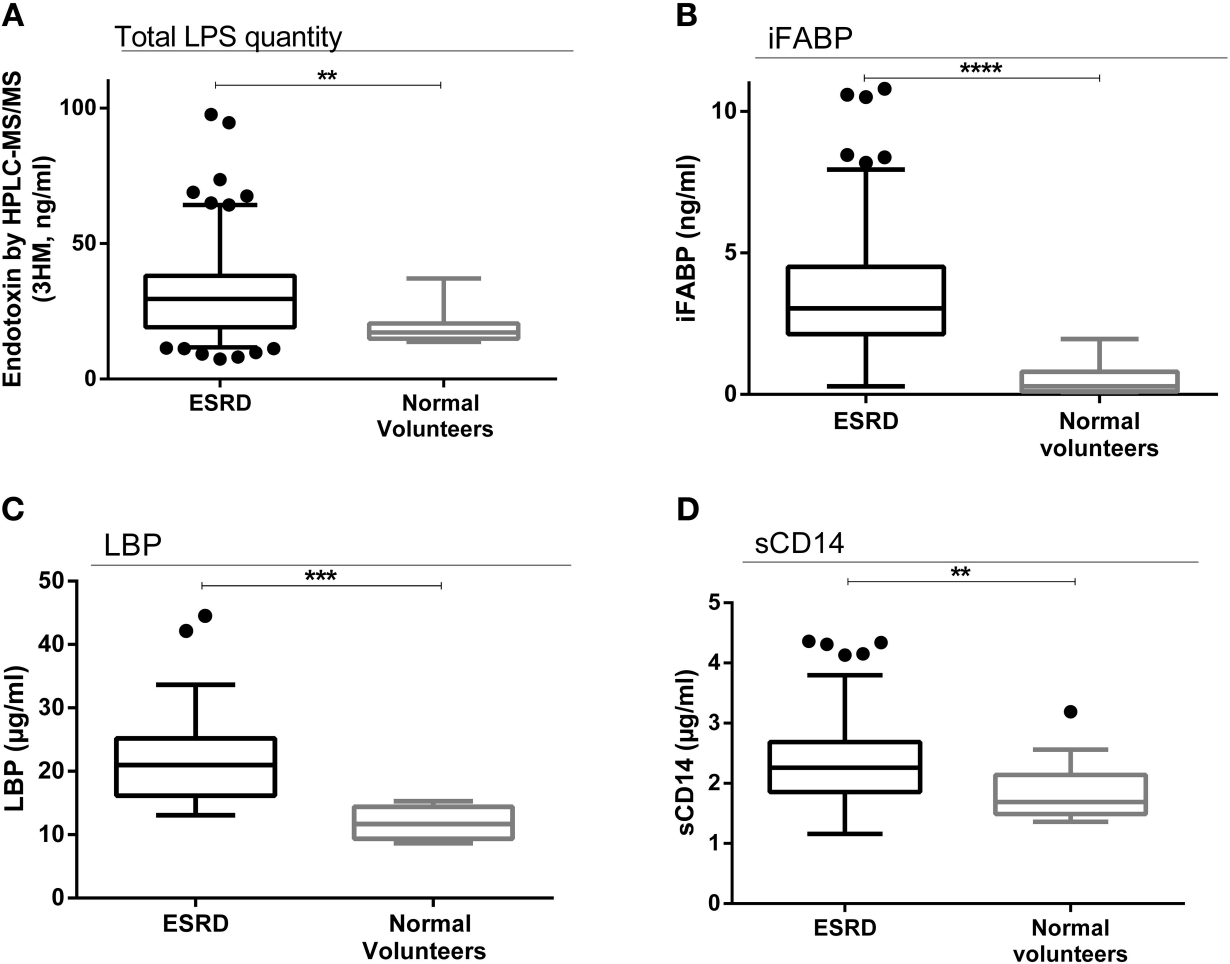
Gut bacterial translocation and chronic inflammation in ESRD population. Comparison of total LPS concentrations between ESRD population (*n* = 146) and normal volunteers (*n* = 11): total LPS concentration was determined in serum of all 146 RTR by direct quantitation of 3-hydroxytetradecanoic acid (3-hydroxymyristate or 3HM) by high performance liquid chromatography coupled with mass spectrometry (HPLC/MS/MS) (16). 3HM is a fatty acid of A lipid, component of LPS. 3-HM molecules are indeed bound to the lipid A motif of LPS and allow us to quantify circulating total LPS (16) **(A)**. Comparison of iFABP concentrations between ESRD population (*n* = 146) and normal volunteers (*n* = 11) **(B)**. Comparison of LBP concentrations between ESRD population (*n* = 57) and normal volunteers (*n* = 11) **(C)**. Comparison of sCD14 concentrations between ESRD population (*n* = 146) and normal volunteers (*n* = 11) **(D)**. Results are expressed in median and 25–75th percentiles using box-and-whisker plots. Only significant *p*-values between groups are represented, **p < 0.01 and ****p < 0.0001.

We first evaluated GBT and inflammation biomarkers in ESRD patients with comparison to those measured in NV. Total LPS concentrations were higher in ESRD patients (mean ± SD: 31.51 ± 15.79 vs. 18.95 ± 6.51 ng 3-hydroxymyristate (3-HM)/ml; *p* = 0.002) (median [interquartile]: 29.59 [19.12–38.13] vs. 17.20 [14.89–20.47] ng 3-hydroxymyristate (3-HM)/ml; *p* = 0.002) (Figure 1A). The concentrations of iFABP were also higher in ESRD patients (mean ± SD: 3.51 ± 2.06 vs. 0.52 ± 0.57 ng/ml; *p* < 0.0001) (median [interquartile]: 3.04 [2.14–4.51] vs. 0.29 [0.11–0.80] ng/ml; *p* < 0.0001) (Figure 1B). Finally, ESRD had an increase in GBT-associated chronic inflammation (LBP: mean ± SD: 21.65 ± 6.57 vs. 11.82 ± 2.72 μg/ml; *p* = 0.0002; median [interquartile]: 20.96 [16.16–25.21] vs. 11.68 [9.36–14.41] ng/ml; *p* = 0.0002) and (mean ± SD: sCD14 levels: 2.34 ± 0.63 vs. 1.89 ± 0.55 μg/ml; *p* = 0.007; median [interquartile]: 2.26 [1.86–2.69] vs. 1.69 [1.49–2.14] μg /ml; *p* = 0.007) (Figures 1C,D). We did not find any correlation between demographics and biomarkers in ESRD.

### Impact of Kidney Transplantation on Gut Bacterial Translocation

Included RTR received more frequently induction therapy with antithymocyte globulins (ATG) (39.0 vs. 29.2%; *p* = 0.042) and were more frequently under azathioprine (2.1 vs. 0.1%; *p* = 0.001) than PNI (Table 1). As expected, we observed an improvement in renal function 1 year post-transplant (Median Glomerular filtration rate estimated by the Modified of Diet in Renal Disease (MDRD) equation = 49 ml/min/1.73 m^2^).

**Figure 2:**
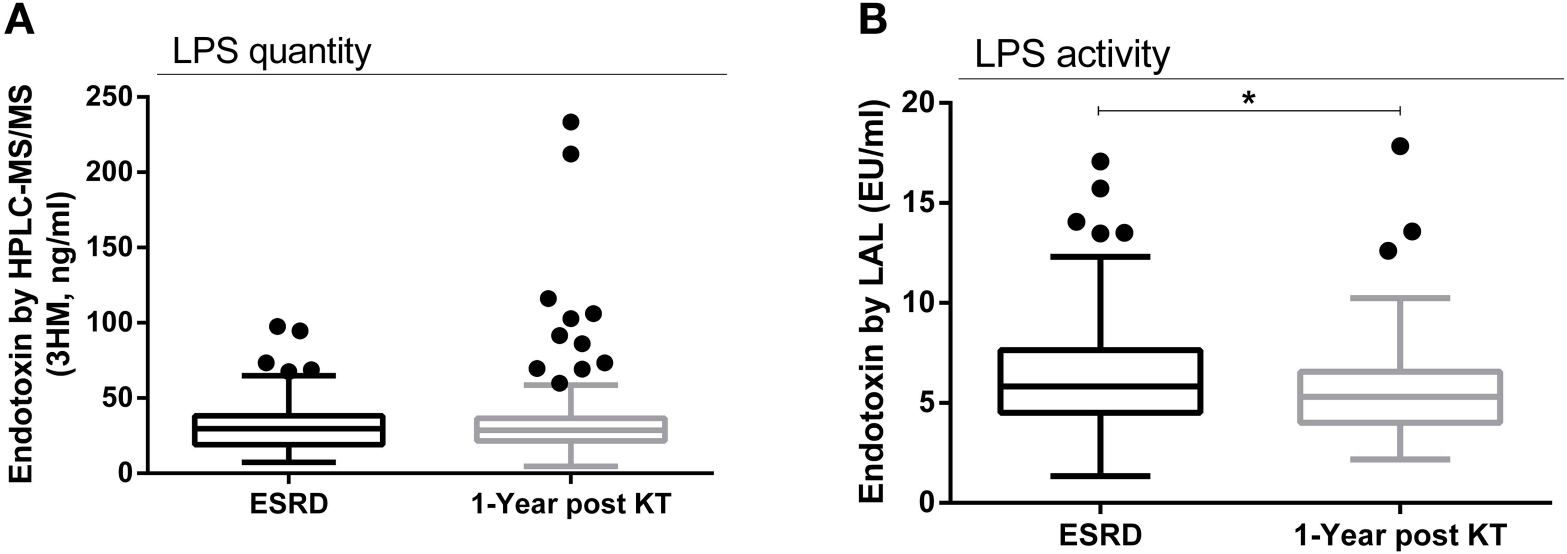
Total LPS quantity and LPS activity prior and 1-year after kidney transplantation. Evolution of total circulating LPS concentrations between ESRD and 1 year after transplantation (*n* = 146): total LPS concentration was determined in serum of all 146 RTR as described in Figure 1 **(A)**. Evolution of the activity of LPS measured by the LAL assay between ESRD and 1 year after transplantation (*n* = 89) **(B)**. Results are expressed in median and 25–75th percentiles using box-and-whisker plots. Only significant *p*-values between groups are represented, *p < 0.05.

Surprisingly, total LPS remained stable without any significant variation 1 year post-transplant (mean ± SD: 31.51 ± 15.79 vs. 34.81 ± 28.57 ng 3HM/ml; *p* = 0.92), (median [interquartile]: 29.59 [19.12–38.13] vs. 28.60 [21.56–36.61] ng 3-hydroxymyristate (3-HM)/ml; *p* = 0.92) (Figure 2A). Hence, improvement in renal function following KT was not associated with a decrease in GBT. Biologically active LPS measured by the LAL assay in NV was not different from ESRD patients (mean ± SD: 6.63±2.03 vs. 6.44±3.04 EU/ml; *p* = 0.46) (median [interquartile]: 6.11 [5.42–7.82] vs. 5.83 [4.50–7.64] EU/ml; *p* = 0.46) (Supplementary Figure 1). Nevertheless, there was a significant decrease in biologically active LPS measured by the LAL assay 1-year post KT (mean ± SD: 6.44 ± 3.04 vs. 5.72 ± 2.47 EU/ml; *p* = 0.03), (median [interquartile]: 5.83 [4.50–7.64] vs. 5.31 [4.01–6.56] EU/ml; *p* = 0.03) (Figure 2B). There was no statistical difference in biologically active LPS measured by the LAL assay between NV and patients at 1-year post-KT (mean ± SD: 6.63 ± 2.03 vs. 5.72 ± 2.47 EU/ml; *p* = 0.07), (median [interquartile]: 6.11 [5.42–7.82] vs. 5.31 [4.01–6.56] EU/ml; *p* = 0.072) (Supplementary Figure 2).

**Figure 3:**
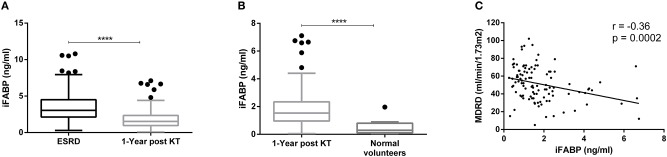
Exploration of gut epithelial barrier integrity by measurement of intestinal Fatty Acid Protein (iFABP). Evolution of iFABP concentrations between ESRD and 1 year after transplantation (*n* = 146) **(A)**. Comparison of iFABP concentrations between renal transplant recipient's population (*n* = 146) and normal volunteers (*n* = 11) **(B)**. Results are expressed in median and 25–75th percentiles using box-and-whisker plots. Spearman correlation between iFABP concentrations and MDRD 1 year after transplantation **(C)**. Only significant *p*-values between groups are represented, ****p < 0.0001.

As LPS quantity remained stable, we analyzed the integrity of the gut epithelial barrier after KT. Interestingly, circulating iFABP concentrations significantly decreased 1 year post-transplant (mean ± SD: 3.51 ± 2.06 vs. 1.90 ± 1.34 ng/ml; *p* < 0.0001) (median [interquartile]: 3.04 [2.14–4.51] vs. 1.53 [0.97–2.35] ng/ml; *p* < 0.0001) (Figure 3A), yet, the titer remained higher when compared to NV (mean ± SD: 1.90 ± 1.34 vs. 0.52 ± 0.57 ng/ml; *p* < 0.0001) (median [interquartile]: 1.53 [0.97–2.35] vs. 0.29 [0.11–0.80] ng/ml; *p* < 0.0001) (Figure 3B). Of note, circulating iFABP concentrations at 1 year post-transplant were inversely correlated with glomerular filtration rate (GFR) (*r* = −0.36; *p* = 0.0002), suggesting a narrow relationship between this biomarker and renal function (Figure 3C).

### Circulating LPS-Induced Inflammation After KT

**Figure 4:**
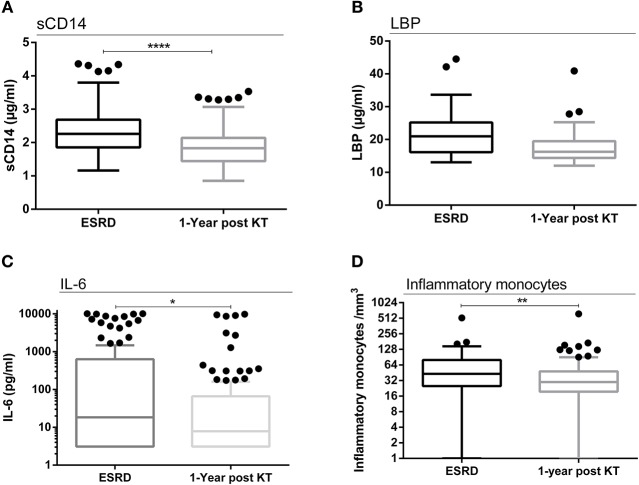
The TLR-4 inflammation pathway. Evolution of sCD14 concentrations between ESRD and 1 year after transplantation (*n* = 146) **(A)**. Evolution of LBP concentrations between ESRD and 1 year after transplantation (*n* = 57) **(B)**. Evolution of IL-6 concentrations between ESRD and 1 year after transplantation (*n* = 89) **(C)**. Evolution of inflammatory monocytes/mm^3^ concentrations between ESRD and 1 year after transplantation (*n* = 74) **(D)**. Results are expressed in median and 25–75th percentiles using box-and-whisker plots. Only significant *p*-values between groups are represented, *p < 0.05, **p < 0.01, ****p < 0.0001.

Because total LPS remained high while bio-active LPS decreased after KT, we explored the LPS-associated inflammation pathway. Circulating sCD14 concentrations significantly decreased 1 year post-transplant (mean ± SD: 2.34 ± 0.63 vs. 1.89 ± 0.54 μg/ml; *p* < 0.0001), (median [interquartile]: 2.26 [1.86–2.69] vs. 1.83 [1.44−2.14] μg /ml; p < 0.0001) (Figure 4A). We observed the same phenomenon for LBP (mean ± SD: 21.65 ± 6.57 vs. 17.72 ± 4.87 μg/ml; *p* < 0.0001), (median [interquartile]: 20.96 [16.16–25.21] vs. 16.26 [14.38–19.47] ng/ml; *p* < 0.0001) (Figure 4B), IL-6 (mean ± SD: 1290 ± 2772 vs. 539.7 ± 1945 pg/ml; *p* = 0.04), (median [interquartile]: 18.36 [3.11–630.40] vs. 7.88 [3.11–66.28] pg/ml; *p* = 0.04) (Figure 4C), and the other pro-inflammatory cytokines (IL-1β: mean ± SD: 631.5 ± 1926 vs. 491.7 ± 2933 pg/ml; *p* = 0.008, median [interquartile]: 3.04 [3.04–165.1] vs. 3.04 [3.04–13.57] pg/ml; *p* = 0.008), (IL-8: mean ± SD: 4108 ± 5392 vs. 2162 ± 4377 pg/ml; *p* = 0.002, median [interquartile]: 812.5 [59.84–7656] vs. 220 [58.54–1624] pg/ml; *p* = 0.002), (TNF-α: mean ± SD: 148.9 ± 362.8 vs. 137.6 ± 497.4 pg/ml; *p* = 0.001, median [interquartile]: 51.86 [31.99–90.85] vs. 38.79 [22.56–53.38] pg/ml; *p* = 0.001). Finally, inflammatory monocyte count (CD45^+^CD14^+^CD16^+^HLA-DR^+^CD86^+^ cells) decreased 1 year post-transplant as well (mean ± SD: 61.61 ± 67.27 vs. 43.70 ± 60.66 /mm^3^; *p* = 0.002), (median [interquartile]: 43[25–80] vs. 30 [19.5–48] /mm3; *p* = 0.002) (Figure 4D). Overall, these data suggest a global decrease in pro-inflammatory mediators after transplantation, despite the persistency of high total LPS concentrations 1 year post-transplant.

### Circulating LPS Neutralization After KT

**Figure 5:**
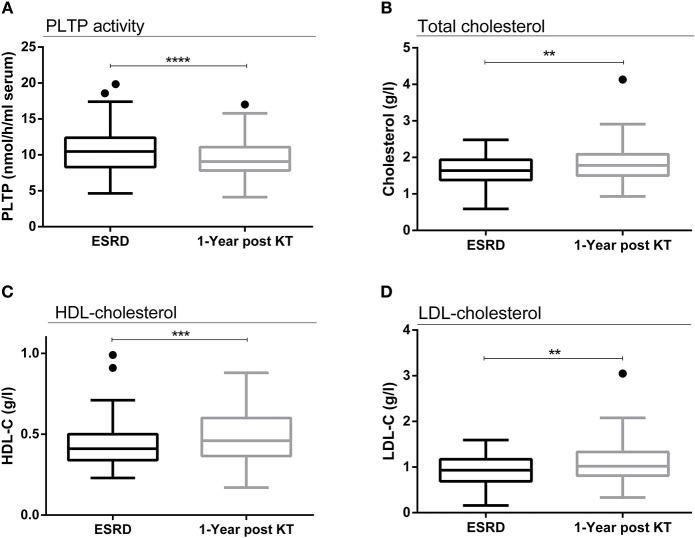
Evolution of PLTP activity, cholesterol and lipoproteins after transplantation. Evolution of PLTP activity between ESRD and 1 year after transplantation (*n* = 89) **(A)**. Evolution of cholesterol concentrations between ESRD and 1 year after transplantation (*n* = 89) **(B)**. Evolution of C-HDL concentrations between ESRD and 1 year after transplantation (*n* = 89) **(C)**. Evolution of C-LDL concentrations between ESRD and 1 year after transplantation (*n* = 89) **(D)**. Results are expressed in median and 25–75th percentiles using box-and-whisker plots. Only significant *p*-values between groups are represented, **p < 0.01, ***p < 0.001, and ****p < 0.0001.

**Figure 6:**
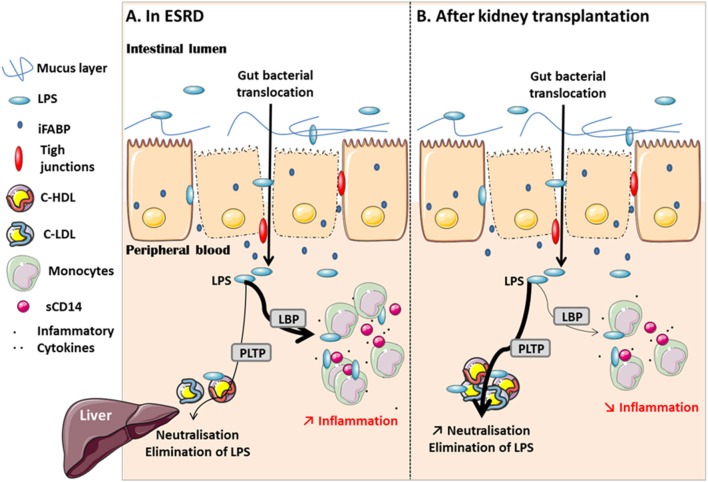
Description of the effect of GBT on inflammation before and after kidney transplantation. In CKD, the disruption of gut barrier allows the translocation of bacterial products (LPS) into peripheral blood. LPS is helped by LBP to promote the development of chronic inflammation. The elimination pathway is limited because of dyslipidemia **(A)**. After KT, the increase of lipoproteins may improve the availability to bind and neutralize LPS. It is associated with a decrease in inflammation 1 year after transplantation **(B)**.

As an explanation of the decrease in the LAL assay and LPS-induced inflammation after KT irrespective of high total LPS concentrations, we hypothesized a better neutralization by the binding of circulating LPS onto carriers. Thus, we analyzed the elimination pathway of LPS through quantification of circulating lipoproteins and measurement of PLTP activity. PLTP activity decreased 1 year post-transplant (mean ± SD: 10.68 ± 3.23 vs. 9.45 ± 2.64 nmol/h/ml serum; *p* < 0.0001), (median [interquartile]: 10.47 [8.30–12.38] vs. 9.07 [7.82–11.07] nmol/h/ml serum; *p* < 0.0001) (Figure 5A). Conversely, there was an increase in total cholesterol (mean ± SD: 1.63 ± 0.38 vs. 1.81 ± 0.50 g/l; *p* = 0.001) (median [interquartile]: 1.64 [1.38–1.94] vs. 1.78 [1.50–2.09] g/l; *p* = 0.001), C-HDL (mean ± SD: 0.43 ± 0.14 vs. 0.48 ± 0.16 g/l; *p* = 0.0002) (median [interquartile]: 0.41 [0.34–0.50] vs. 0.46 [0.37–0.60] g/l; *p* = 0.0002), and cholesterol-low-density lipoprotein (C-LDL mean ± SD: 0.94 ± 0.33 vs. 1.08 ± 0.43 g/l; *p* = 0.004), (median [interquartile]: 0.93 [0.69–1.17] vs. 1.02 [0.81–1.33] g/l; *p* = 0.004) concentrations, (Figures 5B–D). This suggests a higher availability of lipoproteins potentially capable to bind and neutralize LPS (Figure 6).

### Acute Rejection

**Figure 7:**
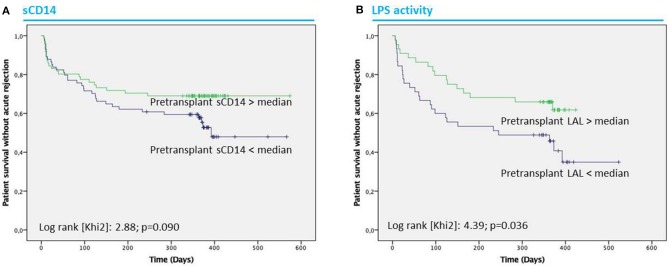
Patient acute rejection-free survival. Acute rejection-free survival according to pre-transplant concentrations of sCD14 separated according to the median (2.26 μg/ml) **(A)**. Acute rejection-free survival according to pre-transplant concentrations of LPS activity measured by the LAL assay separated according to the median (5.83 EU/ml) **(B)**. Statistical analyses were performed by Kaplan Meier test.

Fifty-seven patients (39%) experienced at least one episode of acute rejection (AR) during the first year after transplantation. To test whether circulating LPS or inflammation biomarkers prior to transplantation were associated with AR, we performed a logistic regression including potential factors associated with AR. Univariate analysis (Table 2) revealed that pretransplant sCD14 levels (OR = 0.65 [0.37–1.14], *p* = 0.135), immunosuppressive drugs (MMF: OR = 0.25 [0.08–0.76], *p* = 0.015 and corticoids: OR = 0.29 [0.08–1.01], *p* = 0.051), cytomegalovirus prophylaxis (OR = 0.40 [0.19–0.84], *p* = 0.016), male gender (OR = 0.55 [0.27–1.10], *p* = 0.091), body mass index (OR = 1.05 [0.98–1.12], *p* = 0.152), proportion of effector CD8^+^ T cells (OR = 1.00 [0.98–1.00], *p* = 0.112) and naive CD45RA^+^CD4^+^ T cells (OR = 1.02 [1.00–1.05], *p* = 0.046), delayed graft function (OR = 3.27 [1.41–7.60], *p* = 0.006), NODAT (OR = 2.60 [1.17–5.78], *p* = 0.019), severe bacterial infections (OR = 2.24 [1.11–4.54], *p* = 0.025) and opportunistic infections (OR = 2.06 [1.01–4.21], *p* = 0.048) were associated with AR. In multivariate analysis, sCD14 levels (OR, 0.43 [0.20–0.90], *p* = 0.025), MMF treatment (OR, 0.14 [0.04–0.53], *p* = 0.004), naive CD45RA^+^ CD4^+^ T cell levels (OR, 1.06 [1.02–1.10], *p* = 0.001) were associated with AR. Additionally, we found that pretransplant low sCD14 levels and low values obtained with the LAL assay were associated with lower survival without AR (*p* = 0.090 and *p* = 0.036; Kaplan-Meier test) (Figure 7).

**Table 2:** Association between sCD14 levels and acute rejection: univariate and multivariate analysis.

**Logistic regression, associated factors with acute rejection**		**Univariate analysis**			**Multivariate analysis**		
**Variable at ESRD**	***N***	**OR**	**IC 95%**	***p***	**OR**	**IC 95%**	***p***
Sex	146	0.55	0.27–1.10	0.091			
BMI	143	1.05	0.98–1.12	0.152			
Anti-CMV prophylaxis	142	0.40	0.19–0.84	0.016			
MMF	146	0.25	0.08–0.76	0.015	0.14	0.04–0.53	**0.004**
Corticoids	146	0.29	0.08–1.01	0.051			
% naive CD45RA^+^CD4^+^ T cells	145	1.02	1.00–1.05	0.046	1.06	1.02–1.10	**0.001**
CD8^+^ T cells	145	1.00	0.98–1.00	0.112			
sCD14	145	0.65	0.37–1.14	0.135	0.43	0.20–0.90	**0.025**
**Early clinical events**	***N***	**OR**	**IC 95%**	***p***	**OR**	**IC 95%**	***p***
Delayed graft function	146	3.27	1.41–7.60	0.006			
NODAT	141	2.60	1.17–5.78	0.019	6.36	2.31–17.52	** <0.0001**
Severe bacterial infection	146	2.24	1.11–4.54	0.025	2.31	0.94–5.70	0.068
Opportunistic infection	146	2.06	1.01–4.21	0.048			

*Bold values mean statistically significant values*.

Thus, we considered separately patients according to the median of sCD14 levels in ESRD (2.26 μg/ml) (low and high sCD14 group). Total LPS concentrations were higher in high sCD14 group (34.4 ± 15.9 vs. 28.7 ± 15.9 ng 3HM/ml; *p* = 0.02) whereas the LAL assay data were marginally lower (6.01 ± 2.82 vs. 6.65 ± 3.15 EU/ml; *p* = 0.052). No other differences were observed, especially for inflammatory cytokines which were comparable in both groups. Nevertheless, the rate of AR was marginally higher in the low sCD14 level group than in the high sCD14 level group (24.0% vs. 15.1%; *p* = 0.052).

### Long Term Death-Censored Allograft Survival

The patient group exhibiting a low LPS activity (assessed by LAL assay) at transplant was associated with a significant reduction of death-censored allograft survival (77.4 vs. 93% *p* = 0.041 in High LAL group) (Supplementary Figure 4).

We did not find any influence of sCD14 level on death censored allograft survival or of both biomarkers on patients' death (data no shown).

## Discussion

This is, to our knowledge, the first study exploring the course of ESRD-associated GBT—assessed by quantification of circulating biological activity and total mass LPS levels—and its evolution and impact on transplantation. We confirm gut barrier dysfunction, significant GBT and inflammation in ESRD. The recovery in renal function after KT is associated with better gut integrity, yet this integrity remains pathological when compared to NV. Hence, total circulating LPS does not decrease, yet there is a down-regulation of LPS activity and a decrease in systemic chronic inflammation 1 year post-transplant. The increase in cholesterol and lipoproteins pleads for a higher neutralization capacity of circulating LPS and may contribute to the improvement of ESRD-associated inflammatory status after transplantation (Figure 6).

High serum concentrations of total LPS, iFABP and sCD14 confirm the existence of GBT in ESRD. Kidney transplantation allows the weaning of dialysis and is supposed to decrease uremic toxin concentrations and improve renal and intestinal perfusion (9, 22). In our study, we chose to evaluate the integrity of the gut epithelium barrier with iFABP. This biomarker is a sensitive and specific 14–15 kDa cytoplasmic protein highly expressed in the enterocytes, normally undetectable in serum, but quickly released after intestinal damage before urine secretion (17). High iFABP concentrations could reflect significant gut barrier impairment in CKD. After KT, we observed a significant correlation between iFABP and GFR, suggesting that ESRD could promote its accumulation. Hence, the decrease in iFABP may reflect an improvement in the glomerular filtration of the molecule rather than a real improvement in gut epithelial barrier integrity. Noteworthy, iFABP concentrations after transplantation remain higher compared to NV, which strengthens our interpretation. This may explain why we do not observe a concomitant decrease in total endotoxemia as illustrated by the absence of total LPS variation following KT.

Until now, GBT has been assessed by the identification of the same bacterial species in the intestine microbiota and in the peripheral blood of ESRD patients (23). GBT can also be attested by the LAL assay (measuring bio-active LPS) (24) or by detection of circulating bacterial-derived DNA fragments (*16S rDNA*) (25, 26). Indeed, Shi et al. described the presence of bacteria in the peripheral blood by *16S rDNA* sequencing and suggested that any intestinal bacteria detected in peripheral blood of CKD patients may reveal GBT (25). This hypothesis was later confirmed in dialyzed populations: bacterial species found in dialysates were different from those identified in patients' blood (27), suggesting that the detected species did not translocate from dialysate. The novelty and difference of our study reside in the contemporary measurement of the LAL assay and total circulating LPS by chromatography (16). This latter method sheds new light on the perspectives of GBT exploration and provides new evidence for the intrinsic capacity of circulating lipoproteins and PLTP to neutralize the activity of LPS. Indeed, we observed distinct variations of total LPS measured by chromatography and active LPS measured by the LAL assay. This demonstrates for the first time in KT a persistent GBT with a decrease in circulating LPS activity.

The biological impact of persistent GBT after transplantation seems to be minimized, since we observe a decrease in active LPS associated with a down-regulation of inflammation biomarkers, in particular those related to TLR-4 activation (LBP, sCD14, IL-1β, IL-6, IL-8, and TNF-α). In accordance with the decrease in innate immune pro-inflammatory cytokines, we show a decrease in inflammatory monocyte counts. Nevertheless, it is obvious that inherent anti-inflammatory properties of immunosuppressive drugs used to prevent kidney transplant rejection, especially corticosteroids, calcineurin inhibitors and antimetabolites, could not be excluded from the observed decrease in inflammation. Yet, while inflammatory biomarkers of innate immune system decreased after transplantation, those derived from T helper cell polarization did not decrease (IL-2, IL-10, and IL-15 did not variate and IL-7 and IL-22 increased, data not shown). This could suggest that the down-regulation of the inflammation pathway may be independent from the lymphocyte anti-proliferative effects of maintenance immunosuppressive drugs. Of note, corticosteroids have an impact on monocytes and gene transcription of pro-inflammatory cytokines. This may explain the decrease in inflammation biomarkers of the innate immunity, however, these drugs are mainly used at high doses in the first 3 or 6 months after KT, while we measured these biomarkers 1 year after KT.

Endotoxemia, i.e., accumulation of LPS in blood, may result from the altered intestinal permeability and the ability of neutralization and elimination of circulating LPS by lipoproteins (13). We found higher concentrations of total cholesterol, C-LDL and C-HDL in RTR while the LAL assay and inflammation biomarkers decreased. PLTP activity also decreased 1 year after KT. Although PLTP may accelerate the “reverse LPS transport” pathway, it belongs to a family of lipid transfer/LBP family. Hence, while PLTP activity could be down-regulated like the other inflammation biomarkers, cholesterol, particularly C-HDL (complex structures with surface lipids), are able to bind and detoxify LPS. Accordingly, Galbois et al. showed that incubation of recombinant HDL with whole blood prevented LPS-induced TNF-α and IL-6 overproduction in patients with cirrhosis (28). Dialysis patients present a dyslipidemia with hypertriglyceridemia, elevated lipoprotein-a, low-HDL and reduced apolipoprotein C-I (29, 30). The increase in C-HDL concentrations after KT can be explained by the improvement in the quality of life (31, 32), nutrition status and statin prescription. This increase in lipoproteins could improve binding of circulating LPS, and thus contribute to the improvement of ESRD-associated inflammatory status (33, 34).

We found that elevated sCD14 in ESRD was protective for AR after KT and that lower LAL signal was associated with a better death-censored long-term allograft survival. T and B cells are considered as central effectors in AR, but the role of myeloid cells may be underappreciated (35). Chronic stimulation of myeloid cells by bacterial products has been associated with endotoxin tolerance, a phenomenon whereby prior exposure of organisms or cells to low concentrations of endotoxin causes them to become refractory to further endotoxin stimulation (36, 37). The persistent TLR stimulation induced a subsequent refractory state in which immunostimulation and immunosuppression coexist. Multiple factors were implicated in endotoxin tolerance as dysfunction of lymphocytes and neutrophils; lymphocyte apoptosis; anti-inflammatory cytokine release and monocyte deactivation, rendering them unable to react and perform normal cellular functions (38). Similarly, in CKD/ESRD, the concomitant systemic inflammation and acquired immunodeficiency exist but have not yet been solved (39). In the ESRD high sCD14 level group, even when total LPS was elevated, we observed a lower LAL signal and absence of increase in proinflammatory cytokines. Taken together, these results could suggest endotoxin tolerance in this group. One could extrapolate that repeated exposure to LPS in ESRD reduces the sensitivity to LPS, facilitates suppression of innate and adaptive immunity after KT and prevents AR. This could occur through the induction of regulatory/suppressive immune cell subsets, such as myeloid-derived suppressive cells (MDSC) or regulatory T cells (Treg). We did not have enough samples available to test, MSDSC, anti-donor MLR and anti-donor antibody levels. Yet, proportion of Treg (CD4^+^CD25^high^FoxP3^+^CD127^−^) was available for 54 patients out of 146 patients from a previous work of our group (40). We analyzed the proportion of Treg according to the median of LAL (High or Low LAL group) and sCD14 (High or Low sCD14 group). There was a tendency toward a higher proportion of Treg in High LAL (mean ± SD: 4.68 ± 2.47 vs. 2.44 ± 1.42%; *p* = 0.093; median [interquartile]: 4.0 [2.60–5.50] vs. 2.60 [1.28–3.40] %; *p* = 0.093) and High sCD14 groups (mean ± SD: 3.98 ± 1.17 vs. 3.80 ± 2.62%; *p* = 0.114; median [interquartile]: 4.05 [3.28–4.80] vs. 3.30 [2.40–4.80] %; *p* = 0.114). This result could suggest a degree of endotoxin tolerance in ESRD patients with higher level of translocation biomarkers before transplantation but needs confirmation. Endotoxin tolerance has been reported in several pathologies such as sepsis, cystic fibrosis, acute coronary syndrome, trauma, and pancreatitis (41). In solid organ transplantation, Testro et al. (42) showed that patients who experienced liver AR had higher levels of TLR-4 expression and also greater capacity to produce proinflammatory cytokines following TLR-4 stimulation prior to transplantation. In HIV-infected liver recipients, Balagopal et al. (43) showed that higher log10 sCD14 levels were associated with a 90% reduced risk of graft loss. However, HIV-infected kidney transplant recipients with high levels of immune activation [largely attributed to GBT (7, 44)] were less prone to rejection (45). TLR-4 polymorphism could also play an important role in this phenomenon. Indeed, we previously reported that TRL-4 polymorphisms decreased the risk of AR in RTR (46). Data to support this concept in CKD and KT remain to be determined.

Our study has some limitations. The lack of serum quantity prevented us from performing all assays on all samples. The limited number of included patients and the short-term follow-up did not allow us to better evaluate the long-term clinical impact of GBT. Indeed, McIntyre et al. showed that endotoxemia in ESRD, measured by the LAL assay, is associated with systemic inflammation, features of malnutrition, cardiac injury, and reduced survival (21). Clearly, findings of this preliminary study need now to be validated in larger series. Moreover, we evaluated LPS which is representative of Gram negative bacteria but not Gram positive bacteria. It is generally admitted that Gram-negative facultative anaerobic bacteria and opportunistic pathogens translocate easily than Gram-positive anaerobic bacteria, yet further investigation of intestinal and blood microbiota compositions in RTR remained to be explored (47, 48). Indeed, a disruption in microbiota composition –dysbiosis**-** could promote loss of gut barrier integrity (39) and be one of the causes of persistent GBT after KT. Lee et al. recently showed dysbiosis in RTR (49) and started to evaluate the impact of immunosuppressive drugs on this phenomenon (50). Immunosuppression may have an impact on the gut immunity mucosa (51, 52) and participate to GBT after KT. The evaluation of the impact of each immunosuppressive drug was not possible as most patients had a triple immunosuppression including a CNI. Nevertheless, we did not find any impact of the type of induction therapy (ATG or anti-CD25) (Supplementary Figure 3), or between patients receiving tacrolimus vs. ciclosporine on total or active circulating LPS levels, gut epithelial barrier integrity, lipoproteins and inflammation markers 1 year after transplantation (data not shown). Moreover, in our multicentric study, we did not collect the data concerning the circulating levels of immunosuppressive drugs, and thus, no correlation with GBT can be performed. Finally, LPS has different potential for immune stimulation depending on TLR-4 polymorphism, as well as on the bacterial genus and LPS structure. Vatanen et al. showed that LPS from *Bacteroidetes* possess less immunostimulatory activity than *Escherichia coli* (53), suggesting that all circulating LPS are not equally pro-inflammatory.

Our study is the first to combine total circulating LPS quantification and evaluation of LPS activity to better report GBT *in vivo*. Moreover, it is the first study exploring evolution of GBT after KT. We did not observe any resolution of GBT following transplantation, but rather a better neutralization of total circulating LPS involving increase in cholesterol and resulting in the improvement of GBT**-**associated chronic inflammation. We observed a significant reduced risk of AR after KT in RTR with high pretransplant sCD14 concentration. We hypothesized that LPS repeated exposure in CKD/ESRD patients could promote endotoxin tolerance and immune suppression protecting patients from AR after transplantation. Yet larger prospective cohorts, longitudinal studies and mechanistic data on effectors involved in AR (e.g., anti-donor antibody, Treg, or MDSC analysis) are needed to validate this hypothesis and explore clinical consequences of persistent endotoxemia after kidney transplantation.

## Data Availability

The raw data supporting the conclusions of this manuscript will be made available by the authors, without undue reservation, to any qualified researcher.

## Ethics Statement

Samples collection was performed after regulatory approval by the French ministry of health (agreement numbers # DC-2008-713, June 2009 and DC-2015-2373, October 2015). The ethic committee of Franche-Comté has approved the study (2008). Patients enrolled in the ORLY-EST study gave their written informed consent.

## Author Contributions

CC, DD, PS, and JB participated in research design. CC, J-PP, EG, VD, HA-R, CR, CL, DM, DS-F, PL, BM, LF, PR, CM, AD, A-EH, DD, LL, and JB participated in patients' recruitment and data acquisition. CC, DD, J-PP, VD, PS, LL, and JB participated in data analysis. CC, PS, DD, and JB participated in writing the article.

### Conflict of Interest Statement

The authors declare that the research was conducted in the absence of any commercial or financial relationships that could be construed as a potential conflict of interest.
