# Supplementary material for: End-Stage Renal Disease-Associated Gut Bacterial Translocation: Evolution and Impact on Chronic Inflammation and Acute Rejection After Renal Transplantation
Source: Front Immunol. 2019 Aug 16;10:1630. doi: 10.3389/fimmu.2019.01630 (PMC6706794; doi:10.3389/fimmu.2019.01630)
Supplement: Supplementary file 1 [file Data_Sheet_1.docx]

**Supplementary material**

**Supplementary text**

**Clinical outcomes**

***Post-transplant atherosclerotic events***

Coronary heart disease (CHD): myocardial infarction documented by serial 12-lead electrocardiogram evidence or Q-wave infarction and appropriate myocardial enzyme elevations; coronary revascularization including coronary artery bypass surgery or percutaneous transluminal coronary angioplasty; typical history of angina with abnormal coronarography; sudden death when a history of CHD was present.

Stroke/cerebrovascular disease: nonhemorrhagic strokes confirmed by neurologic examination findings consistent with new onset focal neurologic deficits, with or without computed tomography or magnetic resonance imaging evidence of cerebral infarction; symptomatic extracranial artery stenosis, resulting in carotid endarteriectomy.

Abdominal aortic or lower extremity arterial disease: abdominal aortic repair; lower extremity revascularization via bypass surgery or angioplasty; lower extremity amputation; new onset of intermittent claudication confirmed by Doppler or arteriography findings.

***Acute rejection***

Acute rejection (AR) was considered in the presence of serum creatinine elevation. All episodes of treated AR and biopsy-proven AR were considered separately. Acute rejection was defined according to the Banff classification (1). Systematic biopsies were not performed at any time of the study period.

***Severe bacterial infections***

Diagnosis of severe bacterial infections required bacterial infection-related hospitalization.

***CMV disease***

Diagnosis of CMV disease required the presence of viral replication and a treatment by ganciclovir. (2).

***Opportunistic infections***

Occurrence of opportunistic infections (*Pneumocystis carinii*, tuberculosis, Epstein-Barr virus, toxoplasmosis, aspergillosis, zoster infection, *Legionella pneumophilia*) was recorded.

***New onset diabetes mellitus (NODAT)***

NODAT was defined according to the 2003 International Consensus Guidelines on Diabetes Mellitus in Transplantation (3). This definition is based on the currently accepted criteria proposed by the Canadian Diabetes Association. In summary, diabetes is defined by a casual glucose value of ≥11.1 mmol/L, or a fast plasma glucose (FPG) value of ≥7 mmol/L, or a plasma glucose value of ≥11.1 mmol/L 2 h after a 75-g glucose load. In our cohort, all of the patients with NODAT were identified using FPG. Patients with FPG ≥7 mmol/L or antidiabetic treatment (oral antidiabetic drugs or insulin) were considered to have NODAT.

***Graft Loss***

Graft loss was defined as a continuous recourse to dialysis above a 3 months period.

***Death-censored allograft survival***

Allograft survival was censored for patient death with a functioning graft to estimate the probability of graft loss only. The date of death was considered as the date of last follow-up. The time point of last follow-up was 2019, 8^th^ March.

Two physicians independent of the study and without knowledge of baseline characteristics were responsible for outcomes ascertainment. This analysis was performed without knowledge of baseline characteristics.

**Supplementary bibliography**

1. Haas M, Loupy A, Lefaucheur C, et al. The Banff 2017 Kidney Meeting Report: Revised diagnostic criteria for chronic active T cell-mediated rejection, antibody-mediated rejection, and prospects for integrative endpoints for next-generation clinical trials. Am J Transplant. 2018; 18: 293–307.
2. Courivaud C, Bamoulid J, Chalopin JM, et al. Cytomegalovirus exposure and cardiovascular disease in kidney transplant recipients. J Infect Dis. 2013; 207: 1569-75.
3. Davidson J, Wilkinson A, Dantal J, et al. New-onset diabetes after transplantation: 2003 International consensus guidelines. Proceedings of an international expert panel meeting. Barcelona, Spain, 19 February 2003. Transplantation. 2003; 75(10 Suppl): SS3-24.

**Supplementary tables**

**SuppTable 1: Inclusion and exclusion criteria of the ORLY-Est Study**

| **Inclusion criteria** | **Exclusion criteria** |
| --- | --- |
| Renal Transplant candidates on waiting list (male or female) aged between 18 and 80 y-o | Legal incapacity or legal limited capacity |
| Post menauposal women since at least 24 months, surgicaly sterilised or non menauposal women under effective contraception | Pregnancy |
| Patients who have given an informed consent | Non menauposal women without effective contraception |
| Patients affiliated to the french social insurance or equivalent | Subjects without social insurance |
|  | Subjects currently in a period of exclusion due to enrollment in another study or listed in the national volounteers file |

**SuppTable2: Patients characteristics according to their status for acute rejection**


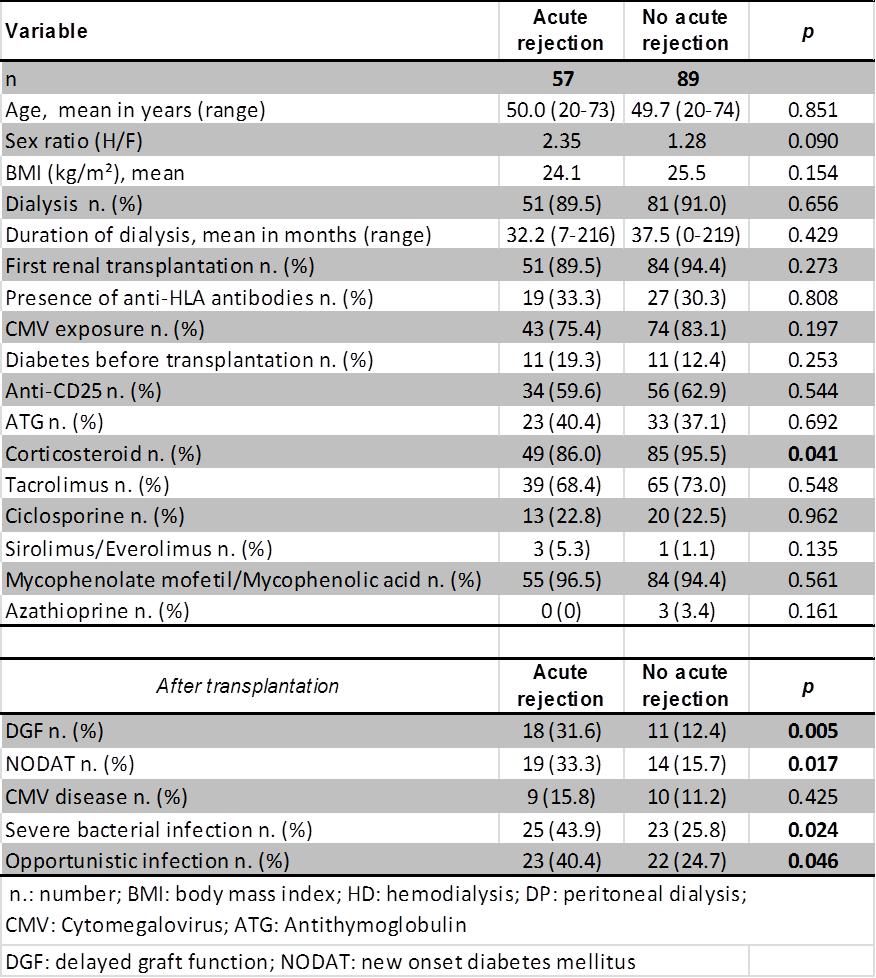


**Supplementary figures**

**Suppfigure 1: Comparison of the activity of LPS measured by the LAL assay between ESRD (n=89) and NV (n=11).**

**Suppfigure 2: Comparison of total circulating LPS concentrations between patients at 1 year-post transplantation and ESRD (n=146) and NV (n=11) :** total LPS concentration was determined in serum of all 146 RTR and 11 NV by direct quantitation of 3-hydroxytetradecanoic acid (3-hydroxymyristate or 3HM) by high performance liquid chromatography coupled with mass spectrometry (HPLC/MS/MS)[15]. 3HM is a fatty acid of A lipid, component of LPS. 3-HM molecules are indeed bound to the lipid A motif of LPS and allow us to quantify circulating total LPS [15].

**SuppFigure 3: Comparison of bacterial translocation biomarkers measured one year post-transplant according to the type of induction therapy received at transplant time** (anti-CD25 (n=90) *vs.* ATG (n=56))**.**

SuppFigure 4: Death censored allograft survival according to pre-transplant concentrations of LPS activity measured by the LAL assay separated according to the median (5.83 EU/ml). Statistical analyses were performed by Kaplan Meier test.
