# Supplementary figures and images for: End-Stage Renal Disease-Associated Gut Bacterial Translocation: Evolution and Impact on Chronic Inflammation and Acute Rejection After Renal Transplantation
Source: Front Immunol. 2019 Aug 16;10:1630. doi: 10.3389/fimmu.2019.01630 (PMC6706794; doi:10.3389/fimmu.2019.01630)

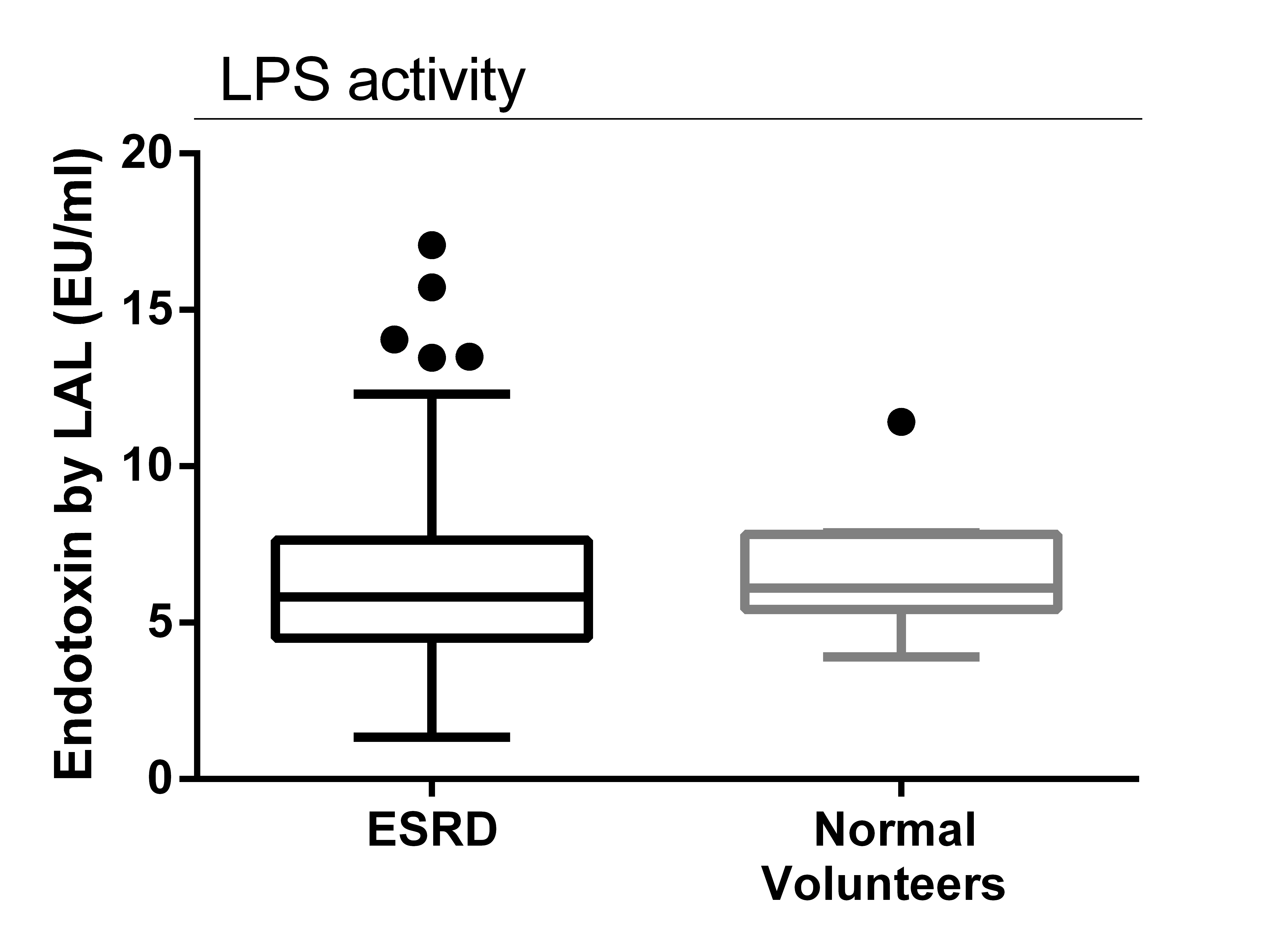

Supplement: Supplementary file 2 [file Image_1.jpg]

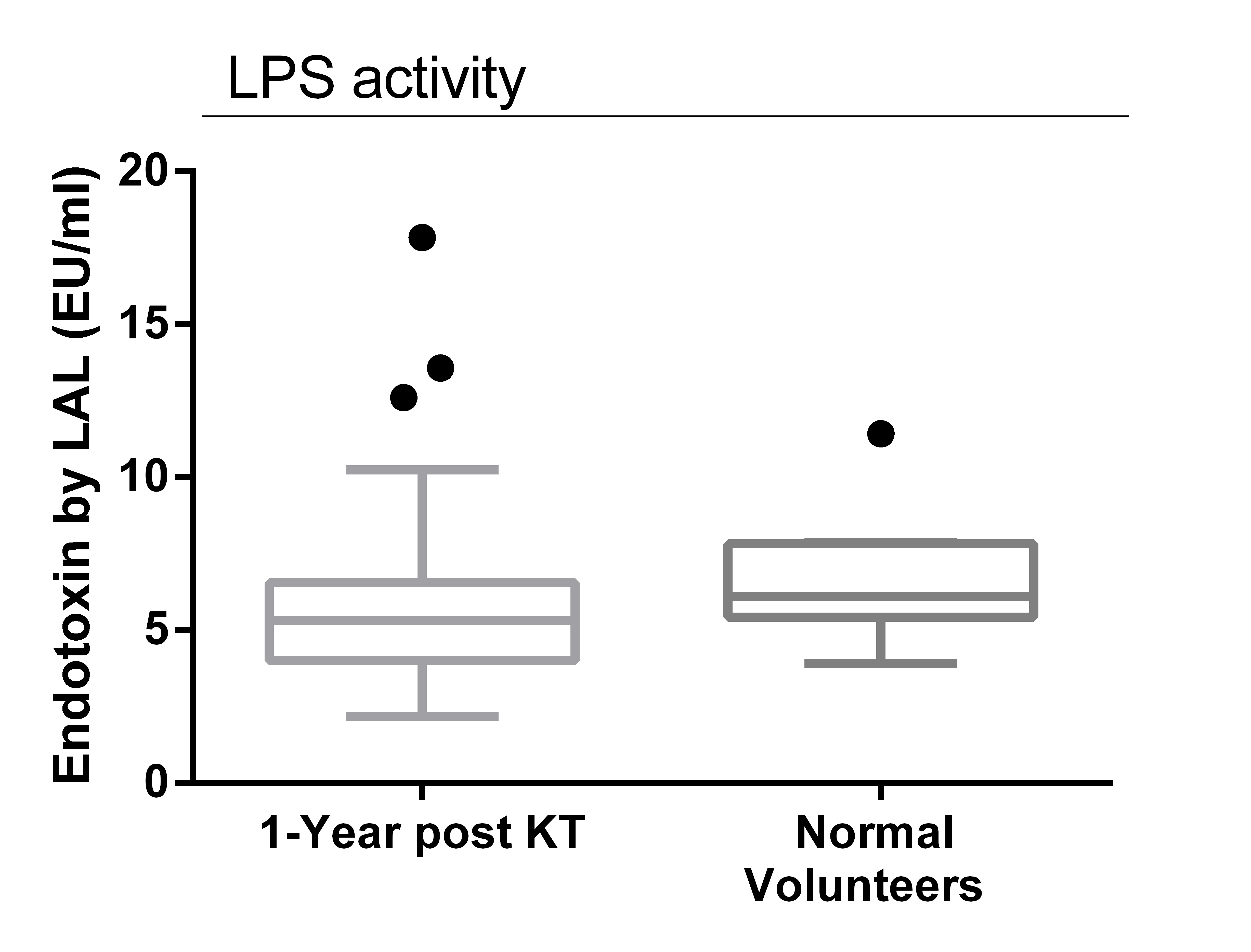

Supplement: Supplementary file 3 [file Image_2.jpg]

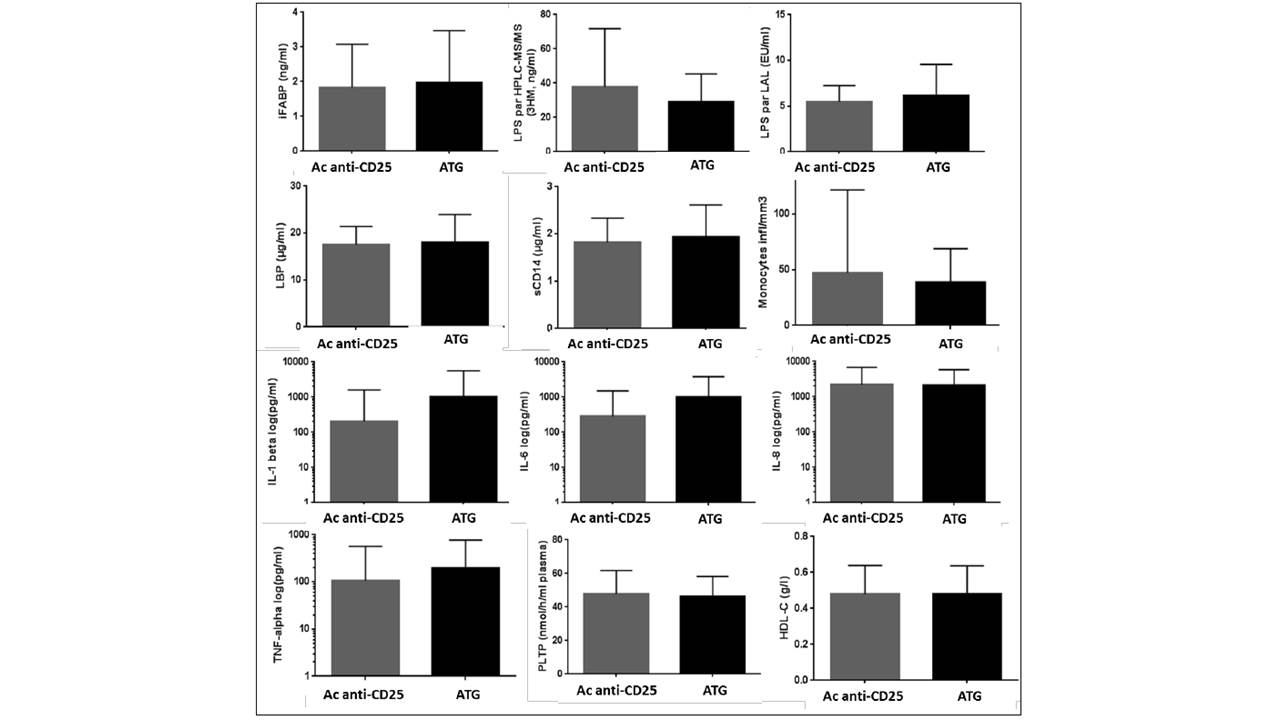

Supplement: Supplementary file 4 [file Image_3.jpg]

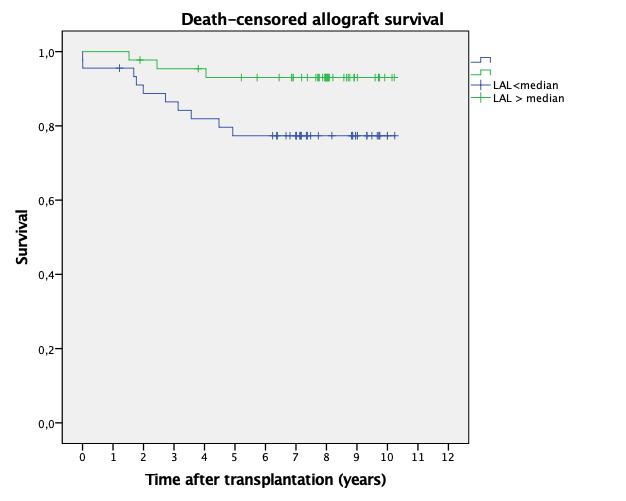

Supplement: Supplementary file 5 [file Image_4.jpg]
